# Routine immunization status of nomadic children aged five years and below in Volta Region, Ghana in the post-COVID-19 pandemic era: a cross-sectional study

**DOI:** 10.1186/s12889-025-23290-2

**Published:** 2025-06-05

**Authors:** Amatus Nambagyira, Samuel Adolf Bosoka, Mavis Pearl Kwabla, Godwin Adjei Vechey, Senanu Kwesi Djokoto, Fortress Yayra Aku

**Affiliations:** 1 Department of Epidemiology and Biostatistics, Fred N. Binka School of Public Health, University of Health and Allied Sciences, Hohoe campus, Hohoe, Ghana; 2 Disease Surveillance Unit, Volta Regional Health Directorate, Ho, Ghana

**Keywords:** Immunization, Nomadic children, Post-COVID-19 pandemic, Fully immunized, Immunization schedule, Volta region, Ghana

## Abstract

**Background:**

Despite the benefits of routine childhood immunization, coverage has remained low in parts of Ghana, particularly among nomadic children. Moreover, the COVID-19 pandemic exacerbated the uptake of routine immunization and other health services. We, therefore, assessed the routine immunization status of nomadic children aged five years and below during the post-COVID-19 pandemic era in two districts of the Volta Region.

**Methods:**

Between July and October 2022, we conducted a community-based analytical cross-sectional study among 157 nomadic children aged five years and below to asses post-COVID-19 pandemic immunization status. Data were collected through interviews of caregivers using a structured questionnaire and analyzed with Stata Version 17. Descriptive statistics were used to summarize the data. A multivariate logistic regression model was used to determine factors associated with full immunization status at *p* < 0.05 and 95% confidence interval.

**Results:**

Of the 157 children involved in the study, males comprised the dominant group, accounting for 52.2% (82/157). The overall complete immunization for age was 51%, with 73.6% full immunization observed among those aged 12–59 months. The odds of full immunization were higher among children aged 24–35 months [aOR = 15.50, 95%CI: (2.03-118.39)] and those aged 36–59 months [aOR = 14.18, 95% CI: (3.17, 63.46)], children of caregivers with a history of postnatal care (PNC) visits [aOR = 4.16, 95% CI: (1.29–13.40)], caregivers being convenient with the immunization schedule [aOR = 4.50, 95% CI: (1.16–17.42)] and those encouraged by community leaders [aOR = 95%CI: (1.06–13.70)]. Caregivers reporting long waiting times at vaccination centres had lower odds [aOR = 0.19, 95% CI: (0.04–0.84)] of full immunization.

**Conclusion:**

The full immunization status of nomadic children under five years in the study area was moderate and was associated with the child’s age, PNC visits, community leader encouragement, the convenience of immunization schedules, and waiting times. We recommend that the District Health directorates employ targeted and multifaceted strategies to address the suboptimal immunization uptake observed among this vulnerable group.

**Supplementary Information:**

The online version contains supplementary material available at 10.1186/s12889-025-23290-2.

## Introduction

The COVID-19 pandemic, declared a Public Health Emergency of International Concern by the World Health Organization (WHO) in March 2020 [1], is widely regarded as the most significant global health crisis of the past century [2]. Over 760 million cases and 6.9 million deaths have been recorded worldwide since December 2019, but the actual number is thought to be higher [1]. Ghana recorded its first two imported cases of COVID-19 on March 12, 2020. Since then, the country has been hit by three major waves. The first wave occurred between June and August 2020 while the second occurred between January and February 2021 [3]. As of 23rd September 2023, Ghana has recorded over 170,000 cases and 1,000 deaths.

The COVID-19 pandemic has disrupted health systems worldwide regardless of income levels [4], and essential healthcare services, including childhood immunization, have been heavily impacted. Globally, an estimated 13.5 million children missed out on routine immunization in 2020 due to the diversion of resources to control the deadly pandemic [5]. Despite WHO recommendations to maintain basic healthcare services, such as maternal and child health (MCH) [6], many countries struggled to sustain the provision of high-quality essential health services during the pandemic [2]. Hence, vulnerable groups, such as the nomadic population, characterized by their highly mobile lifestyles and unique challenges, have faced difficulties accessing these services, including immunization [7, 8]. Moreso, long before the pandemic, children in war-prone areas have also suffered immunization deficits due to conflicts and humanitarian crises [9].

Studies in Sub-Saharan Africa have consistently reported lower rates of childhood immunization among nomadic communities [7, 8, 10–12], with rates as low as 28% in Kenya [10] and 42.2% in southern Ethiopia [12]. In Ghana, there is a dearth of research concerning the immunization status of the nomadic population. Previous studies in this context were conducted in response to outbreaks of vaccine-derived poliovirus type 2 and Yellow fever within the nomadic community [13, 14]. These studies revealed vaccination rates of 80% for yellow fever and 29.6% for poliovirus among nomadic children. However, the overall childhood immunization rate within this population remains unknown, and the factors contributing to full immunization, particularly in the Volta region of Ghana, are not well-defined. Furthermore, the COVID-19 pandemic has exacerbated the uptake of routine immunization and other health services among this vulnerable population, hence the need to assess coverage post the pandemic. This study determined the level of post-COVID-19 pandemic full immunization status and its associated factors among nomadic children under five years of age in two districts of the Volta Region, Ghana.

## Materials and methods

### Study design

We conducted a community-based analytical cross-sectional study in the Adaklu and Akatsi North Districts from July to October 2022.

### Study area and population

The study was conducted in the Adaklu and Akatsi North Districts of the Volta Region of Ghana, among nomadic children aged 5 years and below. The two districts are among the eighteen (18) Municipalities and Districts in the Volta Region of Ghana and have nomadic communities widely dispersed in almost all the sub-districts. Currently, there are seventeen (17) health facilities in Adaklu District (eleven Community-based Health Planning and Services (CHPS) compounds, four health centres and two mission facilities. Akatsi North District has a total of twelve facilities, comprising ten CHPS compounds and two health centres. There is no hospital in the two districts in the region. The main occupations of the populace in the two districts are farming, trading and cattle rearing by the nomads. Both districts have nomadic populations, and it is against this background that the two districts were selected for the study. The study population consisted of nomadic children aged five years and below residing in the Adaklu and Akatsi North Districts and their primary caregivers.

**Fig. 1:**
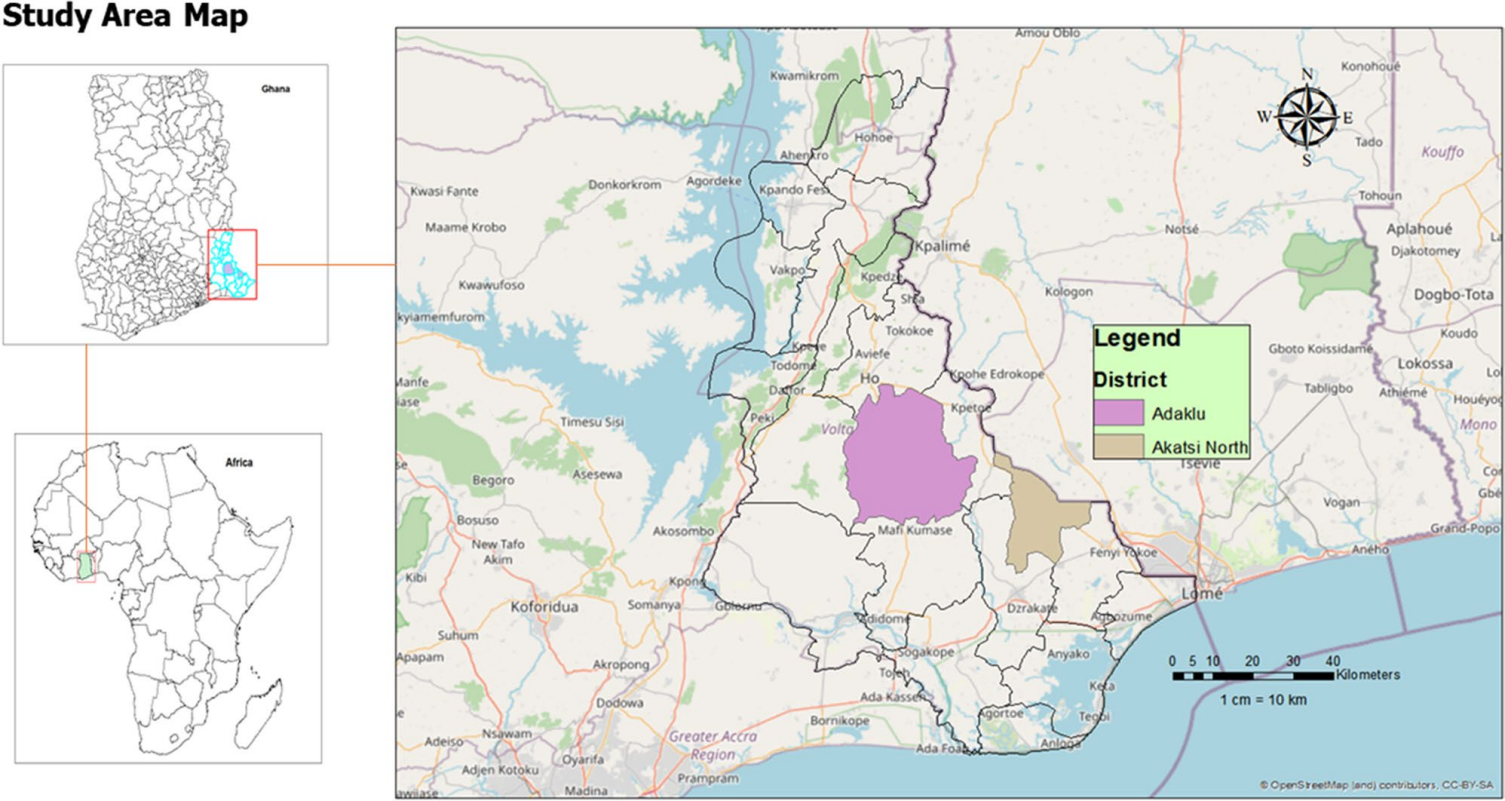
Map showing study areas

Figure 1 illustrates the study site map in the Volta Region of Ghana.

### Sample size determination

The sample size for this study was estimated with the Cochran’s formula [n= (Z² Pq)/d²] [15]. The parameters in the formula include;

n = sample size to be determined.

Z = Reliability of coefficient corresponding to a 95% confidence interval (1.96).

P = proportion of fully immunized children in Ghana was 89.5% [16]. This estimate was used because the study district had similar demographic and health service characteristics with the current study area.

q = the acceptable deviation from the assumed proportion (1-0.895).

d = the margin of error around p estimated as 0.05 in this study.

$$\:\text{n}=\frac{{1.96}^{2}\:\left(0.895*0.105\right)}{{0.05}^{2}}$$

$$\:\text{n}=\frac{{1.96}^{2}\:\left(0.085975\right)}{0.0025}$$

$$\:n\hspace{0.17em}=\hspace{0.17em}144$$

Adjusting for an anticipated 5% non-response rate, a minimum sample size of 157 primary caregivers with nomadic children under five years was recruited for the study.

### Sampling

Adaklu and Akatsi North Districts each consist of five sub-districts, encompassing a total of 104 and 140 communities, respectively. Of these, there are nine nomadic communities in Adaklu and eight in Akatsi North. The initial listing of nomadic communities was purposive based on official and field-level expert identification.

To ensure a systematic and representative sampling process, a multistage sampling technique was employed. This approach resulted in the selection of four sub-districts, twenty communities, and 157 participants from the two districts. The steps taken to achieve this are outlined below:

In Stage 1, sub-districts within each district were randomly selected using the lottery method. A sampling frame of all sub-districts with nomadic populations in each district was created. The names of these sub-districts were written on pieces of paper, folded, and placed in a container. A health professional then randomly selected sub-districts from the container without replacement. Two sub-districts were chosen from each district, resulting in a total of four sub-districts.

Stage 2: A sampling frame of all nomadic communities within the selected sub-districts was created. Five communities were randomly selected from each of the two selected sub-districts in both districts using the lottery method. The names of the communities were written on pieces of paper, folded, and placed in a container. A health professional then randomly selected communities from the container without replacement. Five communities were chosen from each sub-district, resulting in a total of twenty communities. Proportionate sampling ensured each selected community had an equal representative sample size.

Next, in stage 3, in each selected community, data collectors identified a central point such as a market, community center, school, or chief’s palace that served as the geographical center of the community. At this central location, a bottle was spun on the ground to randomly determine a direction in which household selection would begin. Following the direction indicated by the bottle tip, all houses from the central point to the edge of the community along that path were counted and assigned unique sequential numbers. These numbers were then written on pieces of paper, folded, and placed in a container. One number was randomly drawn to identify the first house for data collection.

After the initial household was selected, subsequent households were visited consecutively along that same direction until the required number of eligible participants for the community was obtained. If a selected house had more than one household with eligible participants, one was randomly selected using simple random techniques. If a household did not meet the inclusion criteria or declined participation, the next household was approached.

Finally, in stage 4, the participants were chosen. We recruited a mother or caregiver in each selected household who met the inclusion criteria and consented to participate. In cases where multiple households existed within a house, with eligible children, a simple random selection was done to recruit the participant.

### Data collection instruments and procedures

Two trained research assistants who were fluent in the local dialect (***Ewe***) of the study participants, in addition to the principal investigator, collected data on sociodemographic characteristics, health system, and community-related factors using a questionnaire through interviews. The questionnaire was developed based on insights from the literature reviewed for this study (see Additional File 1). The questionnaire underwent expert consultation through discussions with the district EPI Officers at both study sites to enhance its content validity and ensure alignment with the study objectives. Furthermore, it was subsequently pilot tested among caregivers of children under five years old in a site not included in the main study to identify ambiguous or inconsistent questions before data collection. Before conducting the interviews, each primary caregiver involved in the study provided consent to participate. During the interviews, primary caregivers were asked to present the child’s health record card for review on immunization status. The health record card was used to determine the child’s immunization status. To avoid recall bias, we focused on primary caregivers with health record cards for their children.

### Operational definitions

Based on previous studies [17, 18], we defined immunization status and primary caregivers as follows:

### Fully immunized

For children aged 12–23 months: receiving one dose of BCG, at least three doses of OPV, three doses of pentavalent vaccine, three doses of PCV, two doses of rotavirus vaccine, and one dose of measles vaccines all administered before the child’s first birthday.

For children older than 23 months: receiving one dose of BCG, at least three doses of OPV, three doses of rotavirus vaccines, one dose of measles vaccine, plus a second dose of the measles-rubella (M-R) vaccine and one dose of the meningitis A vaccine.

### Partially immunized

Partially immunized describes a child who has not received one or more of the prescribed vaccine doses based on their age, necessary for safeguarding against vaccine-preventable diseases.

### Not immunized

Not immunized refers to a child who has not received any of the prescribed vaccine doses, which are meant to protect against vaccine-preventable diseases.

### Primary caregivers

A primary caregiver refers to someone who has the main responsibility of caring for the child on a day-to-day basis.

### Data processing and analysis

The data collected was compiled and entered using Epi-Data software version 4.0 and exported into STATA 17.0 for analysis. Data cleaning and validation were done to ensure data quality before analysis. Descriptive statistics, such as frequencies and proportions, were performed on categorical variables, while means and standard deviations were used for quantitative variables and presented in tables and charts. A binary logistic regression model was used to determine the association between the full immunization status of nomadic children (aged 12 months and above) and independent variables. Variables with *p* < 0.2 in the univariate regression were included in the multivariate binary logistic regression. A *p* < 0.05 was considered statistically significant in the final model. For post-estimation, we conducted the Hosmer-Lemeshow goodness-of-fit test to assess the model fit. The test indicated that the model fit the data adequately (p-value > 0.05), suggesting no significant difference between observed and predicted values.

## Results

### Background characteristics of caregivers and children

Of the 157 primary caregivers interviewed, 136 (86.6%) were mothers to the children. The majority, 61.6% (93/157), of the primary caregivers were under 29 years of age, and 91.1% (143/157) were married. Only 19.2% (30/157) of the respondents had basic education and 13.4% (21/157) were livestock farmers. The median age of the children was 28 (23–32) months. Among children aged 13 months and above, 63.7% (100/157) formed the dominant group. A little more than half, 52.2% (82/157), of the children were males, and more than three-quarters, 82.8% (130/157), were delivered at a health facility (Table 1**).**

**Table 1:** Background characteristics of primary caregivers and children under five, Volta region, Ghana

Variables	Frequency (*N* = 157)	Percentage (%)
**Median (IQR) Age of Child in Month**	**28 (23–32)**	
**Age of Child in Month**		
0–11 months	57	**36.3**
12–59 months	100	**63.7**
**Sex of Child**		
Male	82	52.2
Female	75	47.8
**Mean (SD)**	**28.5 (± 7.68)**	
**Age of primary caregivers (years)**		
**≤** 29	93	61.6
**≥** 30	58	38.4
**Relationship with primary caregivers**		
Father	21	13.4
Mother	136	86.6
**Marital status**		
Single	14	8.9
Married	143	91.1
**Education Level**		
No Education	127	80.8
Basic Education	30	19.2
**Religion**		
Christianity	44	28.0
Islam	113	72.0
**Occupation**		
Unemployed	104	66.2
Livestock Farming	21	13.4
Trading	32	20.4
**Birth order**		
≤ 2	64	40.8
≥ 3	93	59.2
**Place of delivery of the child**		
Home	27	17.2
Health facility	130	82.8
**History of PNC attendance**		
No	52	33.1
Yes	105	66.9
**Primary caregiver travel frequency**		
Once every week	41	26.1
Every month	116	73.9

### Complete immunization uptake for age and full immunization status of nomadic children aged below 5 years, Volta region, Ghana

**Fig. 2:**
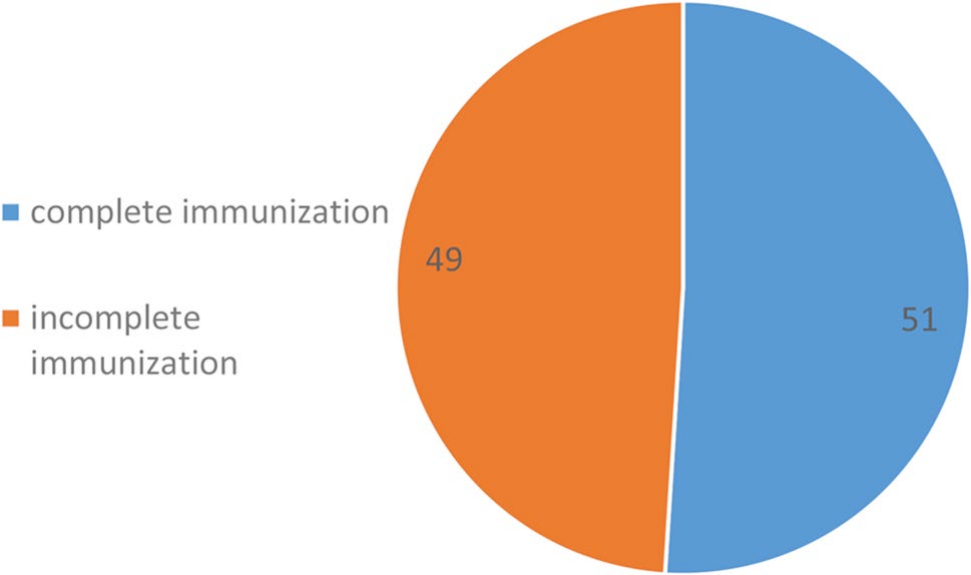
Complete Immunization for age among nomadic children aged below 5 years, in two districts, Volta Region, Ghana

Of the 157 children assessed, we observed that, 51% (80/157) of them had complete immunization for their age, while 49% (77/157) had incomplete immunization for age (Fig. 2).

**Fig. 3:**
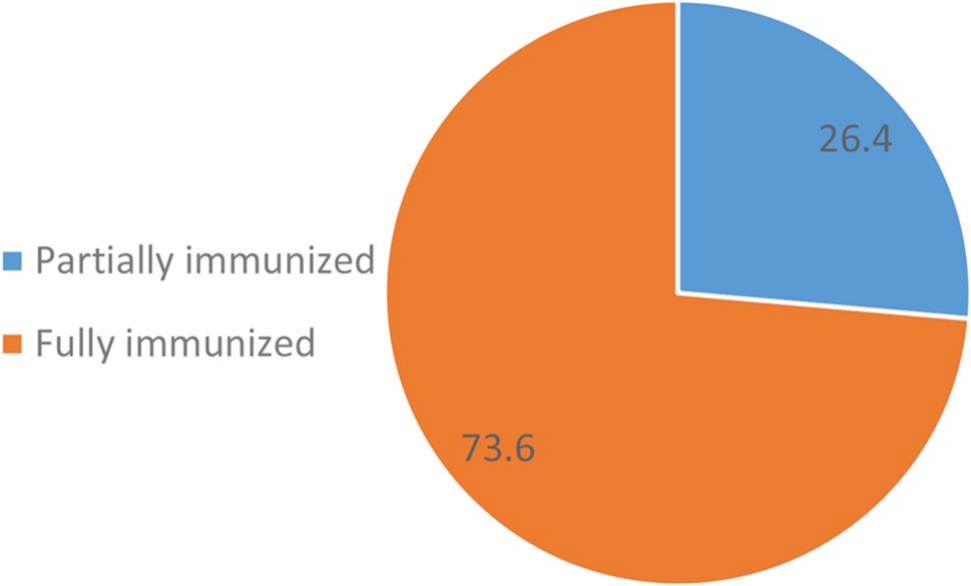
Full Immunization status among nomadic children aged 12–59 months, in two districts, Volta Region, Ghana

Furthermore, among children aged 12 months and above, 73.6% (78/106) were fully immunized while 26.4% (28/106) were partially immunized (Fig. 3).

**Fig. 4:**
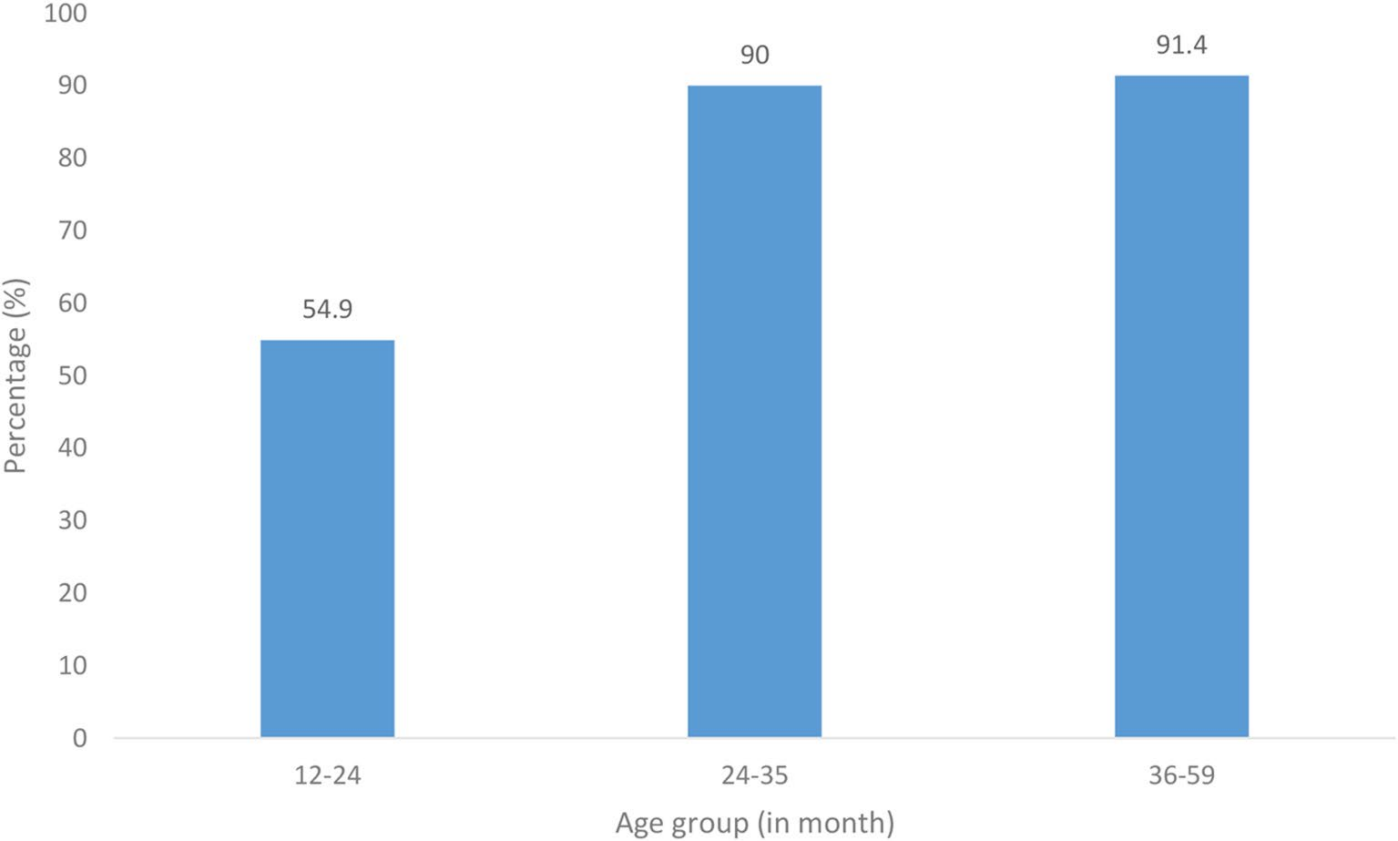
Full immunization status by age among nomadic children aged 12–59 months, in two districts, Volta Region, Ghana

Regarding uptake of full immunization by age, we also found 54.9% (28/51) among children aged 12–23 months, 90% (18/20) among children aged 24–35 months, and 91.4% (32/35) for those aged 36–59 months (Fig. 4).

### Healthcare system factors influencing immunization

Approximately a third, 34.4% (54/157) of caregivers indicated that they could easily access the health facility, while 26.1% (41/157) reported long waiting times at vaccination sessions. The majority, 81.4% (127/157) of the caregivers reported visiting health facilities for other services without vaccination, and 68.8%, (108/157) reported that they were visited in their homes by a healthcare worker (Table 2).

**Table 2:** Healthcare system factors influencing immunization among caregivers of under five years nomadic children in two districts in Ghana

Variables	*N* (%)
**Health facility is easily accessible**	
No	103 (65.6)
Yes	54 (34.4)
**Long waiting at vaccination session**	
No	116 (73.9)
Yes	41(26.1)
**Child developed AEFI after Immunization**	
No	126 (80.3)
Yes	31 (19.8)
**Caregiver at facility without vaccination (missed opportunity)**	
No	29 (18.6)
Yes	127 (81.4)
**Health worker home visit**	
No	49 (31.2)
Yes	108 (68.8)
**Unwillingness of health worker to open new vaccine vial**	
No	128 (81.5)
Yes	29 (18.5)

### Individual-related factors influencing immunization

The majority of caregivers, 77.1% (121/157), reported that immunization schedules were convenient for them, while 28.0% (44/157) reported that they spent money on immunization schedules. More than half, 56.1% (88/157) of the respondents did not need permission from their husbands before sending their children for immunization. Furthermore, 7.6% (12/157) of the respondents indicated that they had witnessed an adverse effect following immunization before, and 66.2% (104/157) of them reported that their community leaders encouraged them to go for immunization (Table 3).

**Table 3:** Individual-Related factors influencing immunization among nomadic children aged under 5 years, Volta region, Ghana

Variable	*N* (%)
**Time and day for immunization is convenient**	
No	36 (22.9)
Yes	121(77.1)
**Spend money on each vaccination session**	
No	113 (72.0)
Yes	44 (28.0)
**Husband permission**	
No	88 (56.1)
Yes	69 (43.9)
**Witnessing AEFI of a child among folks**	
No	145 (92.4)
Yes	12 (7.6)
**Community leader encouragement**	
No	53 (33.8)
Yes	104 (66.2)

### Factors associated with full immunization status of nomadic children

In the bivariate analysis, age of child in months, occupation of caregiver, children of caregivers with a history of postnatal care (PNC) attendance, waiting time at vaccination session, missing vaccination though present at vaccination centre, health worker home visit, caretaker being convenient with immunization schedules, and receipt of encouragement from community leader on immunization were found to be significantly associated with child’s full immunization status (Table 4).

However, after adjusting for confounders in the multivariate model, only child’s age, children of caregivers with a history of postnatal care (PNC) attendance, waiting time at vaccination session, primary caregiver being convenient with immunization schedule, and receipt of encouragement from community leader on immunization were found to be significantly associated with child’s full immunization status.

Children aged 24–36 and 36–59 months were 15 and 14 times more likely to be fully immunized than those aged 12–23 months [Adjusted odds ratio (aOR) = 15.50 (95% CI: 2.03, 118.39) *p* = 0.008], children of caregivers with a history of PNC attendance were 4.16 times more likely to be fully immunized than their counterparts [aOR = 4.16 (95% CI: 1.29, 13.40) *p* = 0.017]. Also, caregivers who reported long waiting times at the vaccination centre had children who were less likely to be fully immunized with a reduced odds of 81 per cent [aOR = 0.19 (95% CI: 0.04, 0.84) *p* = 0.028]. Moreover, caregivers who indicated that healthcare workers visited them at home had children who were 3.42 times more likely to be fully immunized compared to their counterparts [aOR = 3.42 (95% CI: 0.96, 12.22) *p* = 0.058]. However, the difference was not statistically significant. Furthermore, children of caregivers who reported that immunization schedules were convenient for them were 4.5 times more likely to be fully immunized compared to those whose caregivers reported otherwise [aOR = 4.50 (95% CI: 1.16, 17.42) *p* = 0.029. Children of caregivers who received encouragement from community leaders on immunization were 3.8 times more likely to be fully immunized compared to those who did not [aOR = 3.82 (95% CI: 1.06, 13.70) *p* = 0.040] (Table 4).

**Table 4:** Associated factors of full immunization status among nomadic children aged under five years in Adaklu and Akatsi North districts, Volta region

Variable	Immunization status		cOR (95%CI)	*p*-value	aOR (95%CI)	*p*-value
	Partially immunized (*n* = 28) 26.4%	Fully immunized (*n* = 78) 73.6%				
**Age of Child in Month**						
12–23	23 (45.1)	28 (54.9)	Ref.		Ref.	
24–35	2 (10.0)	18 (90.0)	7.39 (1.55, 35.23)	0.012	15.50 (2.03, 118.39)	0.008
36–59	3 (8.6)	32 (91.4)	8.76 (2.37, 32.33)	0.001	14.18 (3.17, 63.46)	0.001
**History of PNC attendance**						
No	15 (45.5)	18 (54.5)	Ref.		Ref.	
Yes	13 (17.8)	60 (82.2)	3.85 (1.55, 9.56)	0.004	4.16 (1.29, 13.40)	0.017
**Long waiting at vaccination session**						
No	20 (22.2)	70 (77.8)	Ref.		Ref.	
Yes	8 (50.0)	8 (50.0)	0.29 (0.10, 0.86)	0.025	0.19 (0.04, 0.84)	0.028
**Health worker home visit**						
No	10 (37.0)	17 (63.0)	Ref.		Ref.	
Yes	18 (22.8)	61 (77.2)	1.99 (0.78, 5.11)	0.151	3.42 (0.96, 12.22)	0.058
**Time and day for immunization is convenient**						
No	8 (40.0)	12 (60.0)	Ref.		Ref.	
Yes	20 (23.3)	66 (76.7)	2.20 (0.79, 6.13)	0.132	4.50 (1.16, 17.42)	0.029
**Witnessing AEFI of a child among folks**						
No	27 (26.5)	75 (73.5)	Ref.		Ref.	
Yes	1 (25.0)	3 (75.0)	1.08 (0.11, 10.83)	0.948		
**Community leader encouragement**						
No	11 (36.7)	19 (63.3)	Ref.		Ref.	
Yes	17 (22.4)	59 (77.6)	2.01 (0.80, 5.03)	0.136	3.82 (1.06, 13.70)	0.040

## Discussion

The current study assessed post-COVID-19 pandemic immunization uptake and its associated factors among nomadic children aged five years and below in the Adaklu and Akatsi North districts of the Volta Region, Ghana. Full immunization status was determined in 73.6% of the children during the study period.

The full immunization uptake observed in our study was higher than the Volta Regional rate of 67.5%, but slightly lower than the national immunization rates of 77.0% [19]. Our rate was lower compared to the findings from previous studies in Ghana. For instance, Djissem et al. [20] reported a 70.0% rate in the Hohoe Municipality, while Wemakor et al. [21] recorded a higher rate of 84.5% in the Kwabre East District of the Ashanti Region. Similarly, Adokiya et al. [16] found an even higher coverage of 89.5% in Techiman. However, our findings were slightly higher than those reported by Meleko et al. [11] in southern Ethiopia (42.2%) and Pertet et al. [21] in a nomadic pastoralist community in Kenya (42%). The general instability of nomadic populations, instances of unmotorable roads leading to their habitation, insufficient outreach activities, and a poor attitude among some health workers towards clients [22, 20] have been identified as barriers to immunization coverage among this population. Strengthening catch-up campaigns, tracking the movement patterns of the nomadic population, integrating routine vaccinations into mass campaigns, and exhibiting good work ethics by health workers could increase coverage [23].

Moreover, since this study was conducted after Ghana’s experience of three waves of the COVID-19 pandemic, with the Volta Region among the top five hit regions with 6,177 confirmed cases, it further raises the urgent need for concerted efforts to improve on immunization coverage among this population [19]. Though studies have generally reported lower immunization coverage among nomadic children before the COVID-19 pandemic, there is the possibility that the gains made have been reversed due to the pandemic [24, 25].

The variation in full immunization rates observed in this present study as compared to previous studies, may be partly attributed to limitations in our sample size. This could have led to either underestimation or overestimation of the true rate. To enhance the accuracy of future research, we recommend that sample size calculations should be guided by the most recent and region-specific immunization estimates.Higher immunization rates among children of caregivers with a history of postnatal care (PNC) visits are consistent with findings by Mekonnen et al. [26] in northwestern Ethiopia among a nomadic population and that of Adetifa et al. [26] in Kenya. Education and counselling received during PNC visits on healthy practices for both mother and child, including favourable outcomes linked to vaccination, could have influenced this observation. Previous knowledge on health choices and outcomes could affect mothers’ subsequent practices, thus immunization.

Furthermore, children whose caregivers reported long waiting periods at vaccination centres were less likely to be fully immunized, which corroborates findings by Ekhaguere et al. [28] and Agócs et al. [27] in Western Kenya. Long waiting periods could connote a loss of productivity for caregivers. On the other hand, it could be attributed to strategies employed by healthcare providers to minimize wastage, such as waiting for a substantial number of children to attend immunization sessions when opening multidose vials. Long waiting periods are particularly applicable to monovalent vaccines in multi-dose vials [27]. When caregivers experience long waiting periods, their economic activities may be affected, particularly for those who belong to the lower wealth quintile, which may demotivate them from adhering to subsequent immunization schedules [28].

In the present study, children whose caregivers reported a healthcare worker home visit were more likely to be fully immunized, however, it was not significant. Conversely, evidence by Boulton et al., [29] in Bangladesh and Shukla [30], in Afghanistan showed home visits by healthcare workers play a significant role in improving immunization uptake as they serve as reminders for the next immunization schedule, while presenting the opportunity to educate caregivers on the importance of child vaccination [21].

As observed in our study, Hoest et al., [31] in a study conducted in eight resource-poor settings (Bangladesh, Brazil, India, Nepal, Peru, Pakistan, South Africa, and Tanzania), reported that, the convenience of the Expanded Programme on Immunization (EPI) schedules for caregivers is a key contributor to a child being fully immunized. Lack of interference in caregivers’ routine schedules is a motivator for their participation in their children’s immunization schedules. Furthermore, we found that community leader encouragement to caregivers increased the odds of vaccination uptake, similar to a previous study in India that, reported positive outcomes on immunization uptake following a community-based intervention which included community awareness raising activities and community guardians offering support during vaccination sessions in the absence of the child’s parents [32]. A post-intervention evaluation of the study conducted in India indicated that community stakeholders’ shared sense of ownership contributed to the observed improvements in immunization uptake. These findings suggest that support from community leaders can play a pivotal role in fostering community engagement and facilitating broader acceptance of health interventions.

### Strengths and limitations

To the best of our knowledge, this is the first study to estimate immunization uptake among nomadic populations in the Volta Region of Ghana. The findings provide valuable baseline data to inform future research, planning, and targeted health service delivery for this often-overlooked population. However, we acknowledge that the sample size in this study is relatively limited and may not be fully representative of the broader nomadic population in the region. This limitation stems from the use of an older immunization coverage estimate derived from a different region to calculate the sample size. As such, caution is advised when generalizing the findings.

We recommend that future studies consider expanding the geographic scope to include more nomadic communities in other districts and use more recent and region-specific data, such as estimates from the 2022 Ghana Demographic and Health Survey (GDHS), to ensure a more representative sample and robust findings.

The cross-sectional design of our study limits our ability to establish a cause-and-effect relationship between full immunization status and its predictors. We also did not collect qualitative data to understand caregivers’ experiences and perspectives on immunization and immunization services. However, our study successfully included a representative sample of nomadic children in the region, allowing for the generalization of the findings to this vulnerable population.

## Conclusion

The study highlights the moderate uptake of routine immunization among nomadic children in the Volta regionand calls for targeted interventions among the younger children and mothers with no previous PNC experience. Health authorities should strengthen home visits and catch-up campaigns and engage nomadic communities towards improving immunization coverage. Since our study is the first among this population, we propose intensified surveillance of immunization indicators by the Expanded Programme on Immunization unit of the Ghana Health Service among nomadic children in the region. Furthermore, we recommend future studies on primary caregivers’ perspectives and experiences of immunization and immunization services to gain a comprehensive understanding of the issue within the regional context.

## Electronic supplementary material

Below is the link to the electronic supplementary material.

Supplementary Material 1

Supplementary Material 2

Supplementary Material 3

## Data Availability

All data supporting the findings of this study are available within the paper.

## Abbreviations

AOR: Adjusted odds ratio
CHPS: Community-based Health Planning and Services
EPI: Expanded Programme on Immunization
MCH: Maternal and child health
PNC: Postnatal care
UHAS-REC: University of Health and Allied Sciences Research Ethical Committee
WHO: World Health Organization
