# Supplementary material for: Routine immunization status of nomadic children aged five years and below in Volta Region, Ghana in the post-COVID-19 pandemic era: a cross-sectional study
Source: BMC Public Health. 2025 Jun 5;25:2098. doi: 10.1186/s12889-025-23290-2 (PMC12139305; doi:10.1186/s12889-025-23290-2)
Supplement: Supplementary file 1 — Supplementary Material 1 [file 12889_2025_23290_MOESM1_ESM.docx]

Supplementary figure 1: Uptake of full immunization among children 12-59 months by district, Volta Region, Ghana
