# Supplementary material for: Routine immunization status of nomadic children aged five years and below in Volta Region, Ghana in the post-COVID-19 pandemic era: a cross-sectional study
Source: BMC Public Health. 2025 Jun 5;25:2098. doi: 10.1186/s12889-025-23290-2 (PMC12139305; doi:10.1186/s12889-025-23290-2)
Supplement: Supplementary file 3 — Supplementary Material 3 [file 12889_2025_23290_MOESM3_ESM.pdf]

Questionnaire

**Topic**	**Associated factors of full immunization status among nomadic children aged five years and below in two districts, Volta Region of Ghana**
**District Name**	
**Sub District**	
**Community**	
**Data Collector's code**	[ ] [ ] [ ]
**Initials of Data Collector**	
**Date**	/ /

Section A: Socio-cultural characteristics

S/N	VARIABLES	RESPONSES
A1	Relationship with the child	1. Father [ ]<br>2. Mother [ ]<br>3. Siblings [ ]<br>4. Others
A2	Age	[ ]
A 3	Sex	1. Male [ ]<br>2. Female [ ]
A 4	Marital status	1. Single [ ]<br>2. Married [ ]<br>3. Separated/ divorced [ ]<br>4. Widow [ ]
A 5	Educational level	1. None [ ]<br>2. Basic [ ]<br>3. SHS [ ]<br>4. Tertiary [ ]
A 6	Religion	1. No religion [ ]<br>2. Christian [ ]<br>3. Muslim [ ]<br>4. Traditionalist [ ]

A 7	Occupation	1. Unemployed [ ]<br>2. Livestock Farming [ ]<br>3. Trading [ ]<br>4. Others [ ]
A 8	Birth order	………………………
A 9	History of ANC attendance	1. Yes [ ]<br>2. No [ ]
A 10	Place of delivery of the child	1. Health facility [ ]<br>2. Home [ ]
A 11	History of PNC attendance	1. Yes [ ]<br>2. No [ ]
A 12	Caretaker or parent travel frequency	1. Everyday [ ]<br>2. Once every week [ ]<br>3. Every month [ ]
A 13	Means of transportation to the vaccination site	1.Walk [ ]<br>2. Motorcycle [ ]<br>3. Car [ ]

Section B: Healthcare system factors influencing immunization

S/N	VARIABLES	RESPONSES
B1	Availability of immunization card (Observe/inspect card for verification)	1. Yes [ ]<br>2. No [ ]
B2	Is the distance to the outreach/facility a challenge (access services)?	3. No [ ]<br>4. Yes [ ]
B3	Do you stay long waiting at vaccination session?	1. No [ ]<br>2. Yes [ ]
B4	Has your child developed any AEFI after the last vaccine received?	1. No [ ]<br>2. Yes [ ]
B5	Did your child get vaccinated when visited the health facility (missed opportunity?	1. No [ ]<br>2. Yes [ ]
B6	Do you pay for your child to get vaccinated?	1. No [ ]

		2. Yes [ ]
B7	Have you been visited by a health worker (home visit) before regarding on vaccination of your child?	1. Yes [ ]<br>2. No [ ]
B8	Have you ever been screamed/shouted at by health worker during vaccination session?	1. Yes [ ]<br>2. No [ ]
B9	Have you ever been reminded on your next visit for vaccination by a health worker?	1. Yes [ ]<br>2. No [ ]
B10	Have you ever visited vaccination session and health worker is unwilling to open a new vaccine vial (unwillingness to open multi-dose vaccine vial)?	1. Yes [ ]<br>2. No [ ]

Section C: Caretaker/parental knowledge influencing immunization

S/N	VARIABLES	RESPONSES
C1	Have you heard vaccination or vaccine preventable diseases?	1. No [ ]<br>2. Yes [ ]
C2	Source of information on vaccination	1. Health worker [ ]<br>2. Friend [ ]<br>3. Radio [ ]<br>4. Other [ ]
C3	Reason for vaccination	1. Protect them from diseases [ ]<br>2. Make them strong [ ]<br>3. Don’t know [ ]
C4	How many vaccines preventable disease do you know?	1. 5 [ ]<br>2. 10 [ ]<br>3. 15 [ ]<br>4. Don’t know [ ]
C5	What age should a child start vaccination?	1. 6 months [ ]<br>2. 1 year [ ]<br>3. At birth/ 0 month [ ]<br>4. Don’t know

C6	At what age should a child complete vaccination?	1. 1 year [ ]<br>2. 3 years [ ]<br>3. 5 years [ ]<br>4. Don't know [ ]

Section D: Community/individual related factors influencing immunization

D1	Are the time and day for outreach/facility vaccination session convenient?	1. Yes [ ]<br>2. No [ ]
D2	Do you spend money on each vaccination session?	1. Yes [ ]<br>2. No [ ]
D3	Does your culture approves/encourages vaccine uptake?	1. Yes [ ]<br>2. No [ ]
D4	Do you need permission from your husband before presenting the child for vaccination?	1. Yes [ ]<br>2. No [ ]
D5	Have you ever been accompanied by your partner/male relative to vaccination session before (male involvement)?	1. Yes [ ]<br>2. No [ ]
D6	Have you ever witnessed any AEFI of a child among your folks?	1. Yes [ ]<br>2. No [ ]
D7	Has any community leader ever encourage you to present your child for vaccination?	1. No [ ]<br>2. Yes [ ]

Section D: Child immunization status (from immunization card)

<table>
<tr><td colspan="6">Child Information</td></tr>
<tr><td>VARIABLES</td><td colspan="5">RESPONSES</td></tr>
<tr><td>Age in months</td><td colspan="5">……………………</td></tr>
<tr><td rowspan="2">Sex</td><td colspan="5">1. Male</td></tr>
<tr><td colspan="5">2. Female</td></tr>
<tr><td rowspan="2">Check from the card if these vaccines have been given</td><td rowspan="2">Unimmunized</td><td rowspan="2">Partially</td><td colspan="3">Fully immunized by age(months)</td></tr>
<tr><td>0-11</td><td>12-23</td><td>>24</td></tr>
<tr><td>BCG</td><td></td><td></td><td></td><td></td><td></td></tr>
<tr><td>OPV</td><td></td><td></td><td></td><td></td><td></td></tr>
</table>

IPV					
PENTA					
ROTA					
PCV					
YF					
MR					
MEN A					
VITAMIN A					
**TOTAL VACCINES**	**……/9**	**……/9**			
